# Complexity of a complex trait locus: *HP*, *HPR*, haemoglobin and cholesterol

**DOI:** 10.1016/j.gene.2012.03.034

**Published:** 2012-05-10

**Authors:** Philip A.I. Guthrie, Santiago Rodriguez, Tom R. Gaunt, Debbie A. Lawlor, George Davey Smith, Ian N.M. Day

**Affiliations:** a Bristol Genetic Epidemiology Laboratory (BGEL), MRC Centre for Causal Analyses in Translational Epidemiology (MRC CAiTE), School of Social and Community Medicine, University of Bristol, Oakfield House, Oakfield Grove, Clifton BS8 2BN, UK; b MRC Centre for Causal Analyses in Translational Epidemiology (MRC CAiTE), School of Social and Community Medicine, University of Bristol, Oakfield House, Oakfield Grove, Clifton BS8 2BN, UK

**Keywords:** *HP*, haptoglobin gene, *HPR*, haptoglobin-related protein gene, LD, linkage disequilibrium, Hb, haemoglobin, CNV, copy number variant, GWAS, genome-wide association study, TC, total cholesterol, LDL-C, low density lipoprotein cholesterol, HDL-C, high density lipoprotein cholesterol, RCC, red cell count, Apo-L, apolipoprotein-L, Apo-A, apolipoprotein-A, SNP, single nucleotide polymorphism, K-EDTA, potassium ethylene diamine tetraacetic acid, ARCS, amplification ratiometry control system, PCR, polymerase chain reaction, HW, Hardy Weinberg, HTR, haplotype trend regression, EM, expectation maximization, PASW, a statistical software package by the company SPSS Inc, PLINK, open-source software for whole genome data analysis, TLF-1, trypanosome lytic factor-1, TLF-2, trypanosome lytic factor-2, kD, kilo Daltons, CHD, coronary heart disease, *HP*, *HPR*, Haemoglobin, Cholesterol, Malaria, Trypanosome

## Abstract

*HP* and *HPR* are related and contiguous genes in strong linkage disequilibrium (LD), encoding haptoglobin and haptoglobin-related protein. These bind and chaperone free Hb for recycling, protecting against oxidation. A copy number variation (CNV) within *HP* (Hp1/Hp2) results in different possible haptoglobin complexes which have differing properties. *HPR* rs2000999 (G/A), identified in meta-GWAS, influences total cholesterol (TC) and LDL-cholesterol (LDL-C). We examined the relationship between *HP* CNV, *HPR* rs2000999, Hb, red cell count (RCC), LDL-C and TC in the British Women's Heart and Health Study (n = 2779 for samples having CNV, rs2000999, and phenotypes). Analysing single markers by linear regression, rs2000999 was associated with LDL-C (β = 0.040 mmol/L, p = 0.023), TC (β = − 0.040 mmol/L, p = 0.019), Hb (β = − 0.044 g/dL, p = 0.028) and borderline with RCC (β = − 0.032 × 10^12^/L, p = 0.066). Analysis of CNV by linear regression revealed an association with Hb (Hp1 vs Hp2, β = 0.057 g/dL, p = 0.004), RCC (β = 0.045 × 10^12^/L, p = 0.014), and showed a trend with LDL-C and TC. There were 3 principal haplotypes (Hp1-G 36%; Hp2-G 45%; Hp2-A 18%). Haplotype comparisons showed that LDL-C and TC associations were from rs2000999; Hb and RCC associations derived largely from the CNV. Distinct genotype–phenotype effects are evident at the genetic epidemiological level once LD has been analysed, perhaps reflecting *HP*–*HPR* functional biology and evolutionary history. The derived Hp2 allele of the *HP* gene has apparently been subject to malaria-driven positive selection. Haptoglobin-related protein binds Hb and apolipoprotein-L, i.e. linking *HPR* to the cholesterol system; and the *HPR*/apo-L complex is specifically trypanolytic. Our analysis illustrates the complex interplay between functions and haplotypes of adjacent genes, environmental context and natural selection, and offers insights into potential use of haptoglobin or haptoglobin-related protein as therapeutic agents.

## Highlights

► *HP* CNV/*HPR* SNP haplotype analysis shows association of *HP* CNV with Hb levels/RCC. ► *HP* CNV/*HPR* SNP haplotype analysis shows association of *HPR* SNP with LDL-C/TC. ► The *HP* CNV/Hb-related association may be via Hp2 allele advantage in malaria zones. ► The *HPR* SNP/cholesterol association is likely via apolipoproteins in TLF-1 and -2. ► We infer that *HP* CNV duplication preceded *HPR* SNP mutation.

## 1 Introduction

The haptoglobin gene exists in the human population in two forms: Hp1 and Hp2. Hp1 is the less frequent allele in Europeans. Exons 5 and 6 of Hp2 represent duplication of a 1.7 kb segment containing exons 3 and 4 of Hp1. The protein product of Hp1,1 is a dimer, whereas the products of Hp1,2 or Hp2,2 are multimers of respectively increasing complexity which have reduced availability to tissues. The function of haptoglobin, which is normally present at greater than a 400-fold molar excess compared with free Hb is to scavenge free Hb which has been liberated into the plasma by intravascular haemolysis. The haptoglobin/Hb complex is cleared from the bloodstream by circulating monocytes or in the liver by Kuppfer cells, and the heme iron is recycled; at sites of tissue damage, the complex is cleared by macrophages. The range of possible forms of haptoglobin, from dimer to large multimer depending on genotype, results in a gene product with a range of properties and potentially complex interactions. For example, although the Hb-binding capacity of Hp2,2 is *lower* than that of Hp1,1 (Okazaki et al., 1997), the Hp2,2–Hb complex binds to CD163 (the haptoglobin receptor expressed on macrophage and monocyte cell surfaces) with a 10-fold *higher* affinity than the Hp1,1–Hb complex (Kristiansen et al., 2001).

There is evidence that the duplication has been shown to be under positive selection in areas where malaria is endemic, with the Hp2,2 genotype being protective (Quaye et al., 2000). Some studies have failed to find protection associated with the Hp2 allele (Bienzle et al., 2005) but this discrepancy may be due to haplotypic association between Hp2 and a haptoglobin promoter SNP present at differing frequencies in the populations studied (Cox et al., 2007). The Hp2 protective effect is likely to have many components, including increased secretion of anti-inflammatory cytokines due to binding of the Hp2,2/Hb complex to the macrophage CD163 receptor (Moestrup and Moller, 2004). The results of association studies between *HP* CNV and LDL-C level have been equivocal (Delanghe et al., 1993; Delanghe et al., 1997; Saha et al., 1992). However, a recent meta-GWAS study (Teslovich et al., 2010) identified a SNP (rs2000999) in the *HPR* gene as marking one of 95 loci influencing lipid levels, and a GWAS study (Igl et al., 2010) identified an association of the same SNP with serum cholesterol levels. We therefore typed both the *HP* CNV and the *HPR* rs2000999 (D′ − 0.91, r^2^ 0.11) in a cohort study (including one with GWAS data available) to test the genotypic association between both genetic variants and LDL-C level. In addition, because of haptoglobin's primary function as a scavenger of free Hb, we set out to test whether there was an association between each polymorphism and Hb concentration, and between each polymorphism and RCC. Haplotypes comprised of *HP* CNV and *HPR* rs2000999 were also constructed and tested in relation to all three traits.

## 2 Materials and methods

### 2.1 *HP* CNV data acquisition

We typed the *HP* CNV in the British Women's Heart and Health Study (BWHHS) (Lawlor et al., 2003), a cohort that originally recruited 4286 women from 23 British towns, aged between 60 and 79 years at enrolment (1999–2001) At baseline assessment the women completed a questionnaire, a nurse-led health interview and a physical examination at which fasting blood samples (requested to fast for a minimum of 8 h) were taken. DNA was extracted from K-EDTA whole blood from 3884 individuals using a salting-out procedure (Miller et al., 1988), and this was analysed using Amplification Ratio Control System (ARCS), a ratiometry-preserving PCR protocol (Guthrie et al., 2011). Briefly, ARCS compares target gene copy number against a single-copy reference gene by amplifying both target and reference using a single universal primer for both ends of both amplicon species, after two initial sequence-specific PCR priming cycles. Amplification with a single universal primer minimises differences in amplification kinetics between target and reference amplicons and ensures the preservation of accurate ratiometry throughout PCR. Numbers of each genotype were obtained as follows: absence of a peak for the *HP* amplicon/presence of a peak for the *TP53* amplicon was classified as Hp1,1; Hp1,2 and Hp2,2 were classified according to their within-cluster positions in scatter plots of fluorescence intensity for *HP* amplicon vs *TP53* amplicon, and in scatter plots of *HP*/*TP53* peak height ratio vs the square root of the sum of the squares of both peaks. Data from DNA which failed to amplify, or whose amplicons fluoresced at an intensity below a minimum threshold, were discarded.

### 2.2 *HPR* SNP data acquisition

*HPR* rs2000999 association data were obtained for the same cohort using the CardioChip 50K SNP genotyping array (Keating et al., 2008), a chip containing 48,000 SNP markers for over 2000 genes associated with cardiovascular disease. Genotyping was successfully performed on 3445 of these samples, and after applying filtering limits for cluster separation (< 0.3), call frequency (< 0.95), mean normalised intensity of the heterozygote cluster (< 0.3), mean of the normalised theta values of the heterozygote cluster (< 0.2 or > 0.8), and heterozygote excess (<− 0.3 or > 0.1), this number was reduced to 3413 (Zabaneh et al., 2011).

### 2.3 Phenotype data acquisition

Haemoglobin and RCC were assayed in local haematology departments in each of the towns within 24 h of the blood being taken, using the standard Coulter counter method on EDTA blood. TC, HDL-C and triglycerides were measured on frozen serum samples using an Hitachi 747 analyser (Roche Diagnostics) and standard reagents. LDL-C was calculated from the Friedewald equation: LDL-C = TC − (HDL-C + triglycerides × 0.45) (Friedewald et al., 1972).

### 2.4 Cohort numbers for CNV and SNP

Of the 4286 participants, 3125 (72.9%) had a valid *HP* CNV genotype and 3436 (80.2%) had valid *HPR* rs2000999 data. Of the 3125 with *HP* CNV, 2887 had complete data on all phenotypes and of the 3436 with *HPR* rs2000999, 3208 had complete data on all phenotypes. Thus, our analyses are conducted on 3125 women when examining associations of *HP* CNV with phenotypes and 3208 women when examining associations of *HPR* rs2000999 with phenotypes. For analyses with haplotypes we have 2779 women.

### 2.5 Statistical analyses

Deviations from Hardy–Weinberg proportions were tested for each marker, using the HW equilibrium calculator (http://www.oege.org/software/hwe-mr-calc.shtml) as previously described (Rodriguez et al., 2009). Analysing single markers, we performed association tests between *HP* CNV/LDL-C and TC levels, and between *HP* CNV/Hb-related phenotypes (Table 1). Similar analyses were performed for *HPR* rs2000999, with the a priori hypothesis that there would be association between both genetic variants and LDL-C, TC, Hb and RCC. Since small allelic effects in complex traits tend to be approximately additive and there is no compelling basis to posit another genetic model for haptoglobin (consider for example Teslovich et al., 2010), linear regression was used to test for association between each phenotype and each genetic marker, using PASW Statistics 18. Haplotypic analyses were conducted using Haplotype Trend Regression (HTR). HTR estimates the haplotype frequencies by use of the expectation–maximization (EM) algorithm and then relates the inferred haplotype frequencies to the observed phenotype using a regression model (Zaykin et al., 2002). The approach gives a significance value (deduced by an F test) and a mean trait value for each haplotype in relation to each trait.

Confidence intervals for phenotypic mean values observed for each haplotype were computed using PASW Statistics 18 from phased haplotypes. Phased haplotypes were derived from PLINK using the command ‘plink–file mydata–hap myfile.hlist–hap-phase’ (Purcell et al., 2007).

Individual level data are subject to data security regulations, but can be provided to any bona fide researcher upon request to the BWHHS committee.

## 3 Results

### 3.1 HP CNV and HPR SNP genotype associations

Allele frequencies for *HP* CNV and *HPR* rs2000999 were in Hardy Weinberg equilibrium (HP CNV *χ*^2^ = 0.32, p = 0.572; HPR rs2000999 *χ*^2^ = 0.62 p = 0.431). Table 1 shows the associations of *HP* CNV and *HPR* rs2000999 with each phenotype. There was no strong statistical evidence of an association between *HP* CNV and LDL-C or TC levels, but there was evidence of association with Hb concentration (p = 0.004) and RCC (p = 0.014); Hp2,2 was associated with the highest Hb concentration and the highest RCC and levels of each phenotype were similar in Hp1,1 and Hp1,2 participants. There was statistical evidence of associations between *HPR* rs2000999 and LDL-C and TC (p = 0.023, p = 0.019 respectively); individuals with either AG or AA had similar levels that were higher than those with GG. rs2000999 also showed association with Hb concentration (p = 0.028); AA was associated with the highest concentration, GG with the lowest and AG intermediate. A trend in the same direction was visible with RCC, though the association failed to reach conventional levels of 5% statistical significance (p = 0.066). The ancestral *HP* non-duplicon allele and ancestral *HPR* rs2000999 genotype GG are both associated with the lowest Hb concentration, together accounting for ~ 0.5% of Hb variance. Similar results were obtained (Supplementary Table 1) when these analyses were repeated considering only those individuals with genotypic data for both markers (n = 2779); in this analysis, power using 2779 participants ranged from ~ 50% to ~ 90%.

### 3.2 Haplotype associations

Of the two locus haplotypes comprised of *HP* CNV and *HPR* rs2000999, one, Hp1-A, occurred at very low frequency (0.6%). The three principal haplotypes are: Hp1-G 36%; Hp2-G 45%; Hp2-A 18%. In Table 2, analysis of haplotypes for LDL-C and TC shows that the Hp2-A haplotype raises the levels of both, the associations derived predominantly from the *HPR* rs2000999 “A” allele (LDL-C β = 0.037 mmol/L, p = 0.011; TC β = 0.036 mmol/L, p = 0.015). Analysis of haplotypes for Hb concentration and RCC shows that the Hp1-G haplotype is associated with lower levels of both, the associations derived mainly from the ancestral *HP* non-duplicon (Hb β = 0.039 g/dL, p = 0.001; RCC β = 0.031 × 10^12^/L, p = 0.010). Table 3, summarising phenotype p-values for different combinations of haplotype comparison, provides strong statistical evidence for cholesterol (TC and LDL-C) level associations with Hp2-G vs Hp2-A, and for Hb-based (Hb level and RCC) associations with Hp1-G vs Hp2-G.

## 4 Discussion

**Fig. 1:**
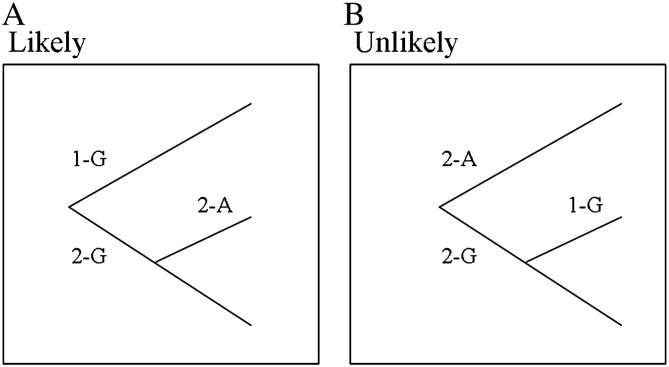
Deduced human evolution for haplotypes of *HP* CNV (alleles 1 and 2) with *HPR* rs200999 (alleles A and G). The frequency of haplotype Hp1-A is extremely low, resulting in effectively three remaining haplotypes. Two cladograms can therefore be considered: panel ‘A’ is the plausible evolutionary route for the emergence of haplotype Hp2-A; panel ‘B’ is difficult to rationalise with the known genomic biology route, since this model requires the non-duplicon Hp1-G haplotype to have arisen from the duplicon haplotype Hp2-G.

**Fig. 2:**
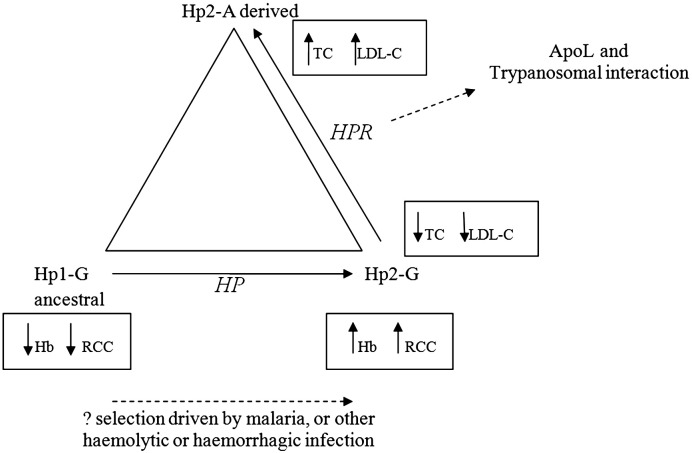
Schematic of haplotype/phenotype interactions. Putative malaria-induced selection pressure has caused an increase in Hb and RCC levels for the Hp1-G to Hp2-G duplication. The inferred Hp2-A derivation from Hp2-G is associated with raised TC and LDL-C levels, possibly reflecting interaction of *HPR* with the lipoprotein system.

Composite analysis of two known important genetic variants in *HP*–*HPR* confirmed an association of the region with serum TC and LDL-C. Also, not previously known, we showed an association between these genetic variants and blood Hb and RCC. We present haplotype data enabling inference of historical order of mutation of these variants (Fig. 1), and enabling inference about mechanisms of causality between the different haplotypes and different phenotypes (Fig. 2).

It is thought that the *HP*–*HPR* gene duplication occurred at least 30 Ma ago (Maeda and Smithies, 1986), with the *HPR* gene, which lies ~ 2.2 kb 3′ to *HP*, arising by non-homologous breakage and reunion. Fig. 1 shows the inferred human evolutionary history of the *HP* CNV and *HPR* SNP variants based on our haplotype analyses. Since there are effectively only three haplotypes, there are two cladograms to consider (Fig. 1 panels A and B). However, if the A/G mutation predated the 1/2 mutation, then a G-1 allele would have had to be formed from a G-2 allele, whereas this is obviously implausible because the non-duplicon allele almost certainly predated the duplicon allele. Therefore Fig. 1A represents the human history of order of these mutations. For markers which are in very close genetic linkage, as these are, it is usual for three haplotypes to be formed (i.e. D′ = 1) and for the fourth only to form later and slowly by recombination events. If A-1 were among the ancestral three haplotypes, then in addition to having rapid recombination to form the fourth haplotype, selection would also have had to eliminate almost entirely, the A-1 allele. Again, therefore, Fig. 1A appears to be the most parsimonious with the data and to represent the likely history.

The relationships between haplotypes and phenotypes are summarised in Fig. 2, in which it can be seen that the *HP* CNV is associated with, and plausibly causal of, effects on Hb and RCC, whereas the cholesterol association relates to *HPR* rs2000999. Known (Atkinson et al., 2007; Quaye et al., 2000) selection pressure (putatively malaria-induced) on haplotypes can be seen to have occurred in conjunction with the occurrence of a haplotype resulting in an increase in Hb and RCC levels. It is plausible that survival in malaria would involve avoidance of extreme anaemia, which even with a small effect such as 0.1 g/dL could give rise to significant selection over a period as short as 2000 years (100 generations). Alternatively, previously reported (Na et al., 2005) anti-oxidation effects of the Hp2 allele may be relevant.

The *HPR* gene is expressed at a level < 10% to that to *HP* (Muranjan et al., 1998; Nielsen et al., 2006), and hence would be expected to exert little effect on Hb levels compared with *HP*. Most of the product of *HPR* is associated with trypanosome lytic factors TLF-1 and TLF-2, both of which contain ApoL-I and ApoA-I. TLF-1 and -2 are both capable of lysing the protozoan parasite *Trypanosoma brucei* (Nielsen and Moestrup, 2009) which causes sleeping sickness in nonprimate mammals, with the *HPR* product acting as a ligand for, and thus guiding the lytic complex to, the parasite surface. *HPR* is therefore linked via TLF-I (containing apoA-I, apoA-II, and apoL-I) to the cholesterol system, thus providing a functional explanation for the *HPR* SNP association. While it is as yet unknown whether *HPR* rs2000999 G/A influences apoL-1 and trypanosome levels, it is known that apoL-1 level is associated with TC (but not HDL-C) and with primary hypercholesterolemia (Duchateau et al., 2000), further supporting the genetic epidemiological evidence pointing to a specific interaction between *HPR* and lipoproteins. However, it remains possible that rs2000999 may proxy a causal variation not in *HPR*, but in *HP* (though not the CNV) or some other neighbouring gene.

The associations we observe between haptoglobin genotype and Hb and RCC fit with known functional biology. Haptoglobin is the principal binding protein for free Hb. Hp2 is associated with a higher Hb and RCC. A previous report showed serum free Hb to be approximately 0.01 g/dL higher in a group of Hp1,1 genotype (Na et al., 2005). This was suggested to reflect the known higher affinity of Hp2,2 for the CD163 receptor on macrophages. Our observation of more than 0.1 g/dL greater Hb in Hp2,2, and a higher RCC, suggests in addition a more efficient recycling process for components of Hb. In any protozoal or parasitic disease causing respectively haemolysis or blood loss, the Hp2 allele would offer a selective advantage, in addition to the reduction of oxidation damage during haemolysis.

Concerning the genetic model (additive, dominant, recessive or intermediate), we used regression tests consistent with the hypothesis of small additive allelic effects typical of complex traits. For *HP* CNV, haptoglobin complexes are largest for Hp2,2, dimers for Hp1,1 and intermediate for Hp1,2. However, the directions of effect on other phenotypes would be difficult to predict from these data alone. For *HPR* rs2000999, there was a prior hypothesis of direction of effect on TC and LDL-C from Teslovich et al. (2010). However, our regression statistic did not incorporate this, although we infer additional significance noting that the allelic direction of effect is the same in our study as in Teslovich et al. (2010). The sample is not large enough to give the statistical power to test the deviation from the additive model across each trait for each genotype. For LDL-C with *HPR* rs2000999, there appears to be a linear pattern across genotypes but less so across CNV genotypes for Hb. It is possible that *HP* CNV may confer nonlinear effects considering that molecular weights have been estimated at ~ 86 kD for genotype Hp1,1; ~ 90 to 300 kD for genotype Hp1,2, and ~ 170 to 900 kD for Hp2,2 (Sadrzadeh and Bozorgmehr, 2004).

From haplotype analysis, it is evident that the cholesterol effects are derived from the *HPR* SNP. Previous reports (Braeckman et al., 1999; Carter and Worwood, 2007; Saha et al., 1992) concerning cholesterol association of the *HP* CNV appear to reflect the linkage disequilibrium of the CNV with another feature in the *HP*–*HPR* region, as yet best marked by the *HPR* rs2000999. By contrast, the Hb and RCC associations, not previously known, reflect the *HP* CNV, which may well be the causal site for that effect. The genetic epidemiological observations appear to mirror the known functional biology, viz. that the product of *HP* is the principal interactor with Hb, whereas the product of *HPR* exhibits specific interactions with the lipoprotein system. These phenotypic effects are of interest with respect to CHD risk, but historically the genetic and haplotype architecture has likely been driven by at least two protozoal infections. The observed haplotypic associations illustrate the complex interplay of genes in blocks of linkage disequilibrium, with different phenotypes relevant to complex traits such as coronary disease. Since haptoglobin is a potential therapeutic product which has been shown to decrease the hypertensive and oxidative effects of Hb in dogs and guinea pigs (Boretti et al., 2009), the genotypic ‘intervention’ may offer useful insight into the possible spectrum of phenotypic changes that might be induced using purified, genetically engineered or pharmacologically induced haptoglobin or haptoglobin-related protein.

### 4.1 Study limitations

The variables chosen were selected from a large number of phenotypes in BWHHS according to our prior hypothesis based on published evidence for *HPR*, and we wanted to apply these results to an analysis of the *HP* CNV. This was therefore an hypothesis-driven selection and in this sense a certain bias cannot be ruled out, but it is clear that a completely open, hypothesis-free screen is an excessive task far beyond the scope of this study.

This study was conducted in a sample of older British women. Cholesterol levels, Hb levels and RCC show age, sex and ancestry differences; it is therefore possible that the observations in this study might not generalise globally. Analyses were conducted on 72.9% and 80.2%, for *HP* CNV and *HPR* rs2000999 respectively, of the original 4286 participants because of missing data. However, it is extremely unlikely that this resulted in any selection bias as participants would have been unaware of their genotype and the genotypic effects on health in earlier life are unlikely to have been large enough to cause survival or ascertainment bias (Rodriguez et al., 2009). Our findings need further replication in other studies.

## 5 Conclusions

Using haplotypes comprised of *HP* CNV and *HPR* rs2000999, we performed association analyses of cholesterol- and haemogobin-related phenotypes in a cohort of British women. We found evidence of association between *HP* CNV and Hb levels/RCC (a novel finding), and between HPR rs2000999 and LDL-C/TC. The *HP* CNV/Hb-related associations are possibly derived from malaria-driven positive selection, mediated through the selective advantage of the Hp2 allele mitigating the deleterious effects of haemolysis and subsequent oxidation damage. The cholesterol associations relate to *HPR* rs2000999, likely through the interactions of the *HPR* product with the lipoprotein system via the trypanosome lytic factors TLF-1 and TLF-2, which contain ApoL-I and ApoA-I. We have inferred the evolutionary history of the *HP* CNV and *HPR* SNP variants in humans, showing that the *HP* CNV duplication event almost certainly preceded the *HPR* SNP G/A mutation event. The haplotypic associations we have observed demonstrate the potential complexity of gene interactions within blocks of LD.

The following are the supplementary materials related to this article.

Supplementary Table 1 As Table 1, but using only individuals with data for *HP* CNV and *HPR* rs2000999.

## Figures and Tables

**Table 1:** Mean levels for LDL-C, TC, Hb and RCC, by *HP* CNV and *HPR* rs2000999 genotype.

	Genotype counts	LDL-C (mmol/L) [SD]	TC (mmol/L) [SD]	Hb conc (g/dL) [SD]	RCC (× 10^12^/L) [SD]
*HP* CNV	2887				
Hp 1,1	394	4.06 [1.05]	6.60 [1.29]	13.46 [1.02]	4.58 [0.39]
Hp 1,2	1326	4.14 [1.08]	6.61 [1.18]	13.49 [1.13]	4.57 [0.39]
Hp 2,2	1167	4.17 [1.12]	6.67 [1.21]	13.62 [1.01]	4.61 [0.37]
p value^a^		0.225	0.112	0.004	0.014

*HPR* rs2000999	3208				
GG	2127	4.12 [1.10]	6.61 [1.23]	13.47 [1.13]	4.57 [0.40]
AG	962	4.20 [1.06]	6.71 [1.19]	13.56 [1.05]	4.59 [0.37]
AA	119	4.25 [1.05]	6.72 [1.09]	13.62 [0.99]	4.64 [0.36]
p value^a^		0.023	0.019	0.028	0.066

RCC R^2^ = 0.1%. All other phenotypes R^2^ = 0.2%.

a p value from linear regression with 1 df.

**Table 2:** Estimated mean levels for LDL-C, TC, Hb, and RCC-haplotype frequencies, confidence intervals and p-values.

Haplotype					
*HP* CNV allele	*HPR* rs2000999 allele	Estimated haplotype frequencies a	Mean LDL-C (mmol/L) b	Confidence interval c	p-value d
Hp1	A	0.0063	3.92	3.37, 4.47	0.570
Hp1	G	0.3621	4.11	4.06, 4.16	0.138
Hp2	A	0.1840	4.22	4.15, 4.29	0.011
Hp2	G	0.4475	4.13	4.09, 4.18	0.632

			Mean TC (mmol/L)		

Hp1	A	0.0063	6.32	5.82, 6.82	0.428
Hp1	G	0.3621	6.61	6.55, 6.66	0.132
Hp2	A	0.1840	6.72	6.64, 6.79	0.015
Hp2	G	0.4475	6.62	6.57, 6.67	0.717

			Mean Hb (g/dL)		

Hp1	A	0.0063	13.53	13.00, 14.05	0.997
Hp1	G	0.3612	13.46	13.41, 13.51	0.001
Hp2	A	0.1835	13.60	13.53, 13.66	0.019
Hp2	G	0.4489	13.55	13.50, 13.59	0.162

			Mean RCC (× 10^12^/L)		

Hp1	A	0.0063	4.56	4.42, 4.70	0.731
Hp1	G	0.3612	4.57	4.55, 4.59	0.010
Hp2	A	0.1835	4.61	4.58, 4.63	0.073
Hp2	G	0.4489	4.59	4.58, 4.61	0.254

a Estimated haplotype frequencies, derived using expectation maximization algorithm with haplotype trend regression (HTR).

b Mean trait value for each haplotype, derived using HTR.

c 95% CI for each trait value from phased haplotypes.

d p-value for association between haplotype and trait value, derived using HTR.

**Table 3:** p-values of inter-haplotype comparisons for mean LDL-C, TC, Hb, and RCC.

Haplotype interaction	LDLC (mmol/L) p-value^a^	TC (mmol/L) p-value	Hb conc (g/dL) p-value	RCC (× 10^12^/L) p-value
Hp2-G vs Hp2-A	0.033	0.036	0.192	0.321
Hp1-G vs Hp2-G	0.551	0.708	0.041	0.010
Hp1-G vs Hp2-A	0.009	0.021	0.011	0.001

a p value is generated by linear regression of haplotypes against each phenotype.
