# Supplementary material for: Complexity of a complex trait locus: HP, HPR, haemoglobin and cholesterol
Source: Gene. 2012 May 10;499(1):8–13. doi: 10.1016/j.gene.2012.03.034 (PMC3405512; doi:10.1016/j.gene.2012.03.034)
Supplement: Supplementary Table 1 — As Table 1, but using only individuals with data for HP CNV and HPR rs2000999. [file mmc1.doc]

**Supplementary Table 1-** As Table 1, but using only individuals with data for *HP* CNV and *HPR* rs2000999

|  | LDL-C  (mmol/L) [SD] | TC  (mmol/L) [SD] | Hb conc  (g/dL) [SD] | RCC  (x 10^12^/L) [SD] |
| --- | --- | --- | --- | --- |
| HP CNV  (n =2779) |  |  |  |  |
| Hp 1,1 | 4.04 [1.05] | 6.58 [1.31] | 13.43 [1.03] | 4.57 [0.39] |
| Hp 1,2 | 4.15 [1.08] | 6.62 [1.19] | 13.48 [1.13] | 4.57 [0.39] |
| Hp 2,2 | 4.17 [1.14] | 6.67 [1.22] | 13.61 [1.00] | 4.61 [0.37] |
| p value | 0.097 | 0.167 | 0.001 | 0.009 |
|  |  |  |  |  |
| HPR rs2000999  (n =2779) |  |  |  |  |
| GG | 4.10 [1.10] | 6.60 [1.23] | 13.49 [1.08] | 4.58 [0.39] |
| AG | 4.20 [1.09] | 6.70 [1.21] | 13.58 [1.05] | 4.60 [0.37] |
| AA | 4.27 [1.08] | 6.75 [1.12] | 13.64 [0.99] | 4.64 [0.35] |
| p value | 0.016 | 0.027 | 0.018 | 0.072 |
